# Association between skeletal muscle mass or percent body fat and metabolic syndrome development in Japanese women: A 7-year prospective study

**DOI:** 10.1371/journal.pone.0263213

**Published:** 2022-10-06

**Authors:** Yosuke Yamada, Haruka Murakami, Ryoko Kawakami, Yuko Gando, Hinako Nanri, Takashi Nakagata, Daiki Watanabe, Tsukasa Yoshida, Yoichi Hatamoto, Eiichi Yoshimura, Kiyoshi Sanada, Nobuyuki Miyatake, Motohiko Miyachi

**Affiliations:** 1 National Institute of Health and Nutrition, National Institutes of Biomedical Innovation, Health and Nutrition, Tokyo, Japan; 2 Faculty of Sport and Health Science, Ritsumeikan University, Kusatsu, Shiga, Japan; 3 Faculty of Sport Sciences, Waseda University, Tokorozawa, Saitama, Japan; 4 Faculty of Sport Science, Surugadai University, Hanno, Saitama, Japan; 5 Department of Hygine, Faculty of Medicine, Kagawa Unviersity, Miki, Kagawa, Japan; UAB School of Medicine, UNITED STATES

## Abstract

Previous cross-sectional studies have indicated that low relative appendicular lean mass (ALM) against body weight (divided by body weight, ALM/Wt, or divided by body mass index, ALM/BMI) was negatively associated with metabolic syndrome (MetS). Conversely, previous cross-sectional studies have indicated that the absolute ALM or ALM divided by squared height (ALM/Ht^2^) were positively associated with MetS. The aim of this longitudinal study was to investigate the association between low absolute or relative skeletal muscle mass, leg muscle power, or percent body fat and the development of MetS in Japanese women in a 7-y prospective study. The study participants included 346 Japanese women aged 26 to 85 years. The participants were divided into low and high groups based on the median values of ALM/Wt, ALM/BMI, ALM/Ht^2^, absolute ALM, or leg power. The longitudinal relationship between ALM indices or leg power and MetS development was examined using Kaplan-Meier curves and Cox regression models (average follow-up duration 7 years, range 1 to 10 years). During follow-up, 24 participants developed MetS. MetS incidence was higher in the low ALM/Wt group than the high ALM/Wt group even after controlling for age, obesity, waist circumference, family history of diabetes, smoking, and physical activity [adjusted hazard ratio = 5.60 (95% CI; 1.04–30.0)]. In contrast, MetS incidence was lower in the low ALM/Ht^2^ group than the high ALM/Ht^2^ group [adjusted hazard ratio = 10.6 (95%CI; 1.27–89.1)]. MetS incidence was not significantly different between the low and high ALM/BMI, absolute ALM, and leg power groups. Both ALM/Ht^2^ and ALM/Wt were not significant predictive variables for MetS development when fat mass or percent body fat was taken into account in the Cox model. At the very least, the results of this study underscore the importance of body composition measurements in that percent body fat, but not ALM, is associated with MetS development.

## 1. Introduction

The decrease in skeletal muscle mass (SMM) and its function are considered important biomarkers of aging [1–3]. Age-related loss of SMM and its function is also known as sarcopenia [4]. SMM can be calculated through appendicular lean mass (ALM) [5–7]. The current measureable definition of sarcopenia is based on ALM and grip strength and/or lower body physical performance [1, 2]. Skeletal muscles are a metabolically active organ that mediates energy metabolism and exerts beneficial effects on metabolic health [8, 9]. Thus, higher muscle mass or muscle function might have a beneficial effect on preventing metabolic syndrome (MetS) [10–14]. MetS is a cluster of conditions that occur together, increasing the risk of heart disease, stroke, and type 2 diabetes. These conditions include visceral fat accumulation, increased blood pressure, dyslipidemia, and hyperglycemia.

However, people with a higher body weight exhibit higher muscle mass and strength, and previous studies indicated that the normalization of muscle mass or strength by body weight or body mass index (BMI) is needed to see the association between SMM or strength and MetS [10–17]. Previous cross-sectional studies indicated that relative ALM per kg weight (ALM/Wt) or BMI (ALM/BMI) are inversely associated with MetS [10–17], but relative ALM (or fat-free mass) per squared height (ALM/Ht^2^) or absolute ALM (or fat-free mass) are positively associated with MetS [18–21].

An important fact is that ALM (or fat-free mass) and fat mass (or percent body fat) are positively correlated with each other and can be confounding factors. We hypothesized that fat mass or percent body fat affects the association between ALM and MetS development. The present longitudinal study aimed to examine the association between ALM, leg muscle power, or percent body fat and MetS development in Japanese women.

## 2. Materials and methods

### 2.1. Ethics approval and consent to participate

The study was performed in accordance with the guidelines of the Declaration of Helsinki. All procedures were reviewed and approved by the ethics committees of the National Institutes of Biomedical Innovation, Health, and Nutrition (6008, Kenei 14–02). All participants provided written consent for participation in the study. The study was performed in accordance with the guidelines of the Declaration of Helsinki.

### 2.2. Participants

This is a secondary analysis of the existing cohort study [16, 22–24]. In this study, we enrolled female participants aged 20 or older who underwent comprehensive health examinations annually at the National Institute of Health and Nutrition, Tokyo, Japan. From a total of 760 women, 346 women aged 26 to 85 years old (mean and SD of age, ± years) were included in the current study upon meeting the following criteria: (1) They received anthropometric and physical activity measurements. (2) Underwent blood examinations. (3) Dietary intake assessments. (4) No history of MetS at the baseline measurement. (5) Underwent follow-up examinations. The baseline measurement was conducted between March 2007 and March 2014. Final measurement was conducted in January 2018. The average follow-up duration was 7 years with a range of 1 to 10 years.

### 2.3. Anthropometric and leg power measures

Before anthropometric measurement, the participants were requested to undertake an overnight fast (>10 h) and to refrain from vigorous exercise and alcohol intake for 24 h. All anthropometric measurements were performed between 9:00 and 10:30 AM. Height was measured using a standard stadiometer to the nearest 0.1 cm. Weight was measured to the nearest 0.1 kg, and body composition was estimated using a segmental, 50 kHz single-frequency bioelectrical impedance analysis (TANITA BC-600). The validity of TANITA BC-600 has been described previously [23]. TANITA BC-600 was used to estimate arm and leg lean soft tissue mass. ALM was calculated as the sum of arm and leg lean soft tissue mass. Waist circumference was measured following a WHO protocol indicating that the measurement be made at the approximate midpoint between the lower margin of the last palpable rib and the top of the iliac crest using a tape measure to the nearest 0.1 cm [25]. ALM/Wt (%) was calculated as ALM divided by body weight × 100 (%). ALM/BMI and ALM/Ht^2^ were also calculated.

Leg extension power was measured by using a dynamometer (Anaero Press 3500; Combi Wellness, Tokyo, Japan) in the sitting position [26]. Device details have been described previously [27, 28]. The participants were advised to vigorously extend their legs. A total of 5 trials were performed at 15-s intervals, and the average of the 2 highest recorded power outputs (in W) was taken as the definitive measurement. The leg extension power divided by body weight was obtained.

### 2.4. Physical activity

The duration and intensity of physical activity were evaluated using a triaxial accelerometer (Actimarker EW4800; Panasonic, Osaka, Japan) [29, 30], as described previously [26]. Participants were asked to wear the physical activity monitor on their hip for 28 days; we used data from 14 days, during which the accelerometer was worn continuously from the time the participant awoke until they went to bed. Physical activity level was obtained as previously described [29, 30].

### 2.5. Blood samples

Blood samples were taken from participants following an overnight fast of at least 10 h [22]. Venous blood withdrawn from the antecubital vein was collected into tubes without additives or EDTA and was immediately centrifuged at 3000 rpm for 20 min to obtain serum or plasma. The levels of glucose, HbA1c, homeostasis model assessment of insulin resistance (HOMA-IR), and HOMA-β in plasma and total cholesterol, high-density and low-density lipoprotein (HDL and LDL) cholesterol, and triglycerides in serum were measured or determined using standard procedures at LSI Medience Corporation (Tokyo, Japan) [26].

According to the definition released by the Japanese Committee for the Diagnostic Criteria of Metabolic Syndrome in April 2005 [31], we defined MetS as the presence of 2 or more abnormalities in addition to visceral obesity (waist circumference: 85 cm or more in men, 90 cm or more in women). These three abnormalities are as follows: 1) Triglycerides ≥150 mg/dL and/or HDL-cholesterol <40 mg/dL or under treatment for this type of dyslipidemia. 2) Systolic blood pressure ≥130 mmHg and/or diastolic blood pressure ≥85 mmHg, or under treatment for hypertension. 3) Fasting glucose ≥110 mg/dL or under treatment for diabetes [31].

### 2.6. Statistical analysis

The results are presented as means ± SD. Differences were analyzed using ANOVA. Cumulative event rates for MetS incidence were estimated using Kaplan-Meier curves, and the equalities were compared using the log-rank test. Cox proportional hazard analysis was performed to determine the independent association between baseline SMI, ALM/BMI, or leg power against other variables. For multivariate analysis, model 1 was a crude form; age, obesity (BMI ≥25 kg/m^2^), and waist circumference were adjusted for in model 2; model 3 included model 2 adjustment and family history of diabetes, smoking status, and physical activity level. Alpha of 0.05 was employed to denote significant statistical deviation. We performed all analyses using IBM SPSS Statistics for Windows, version 22.0 (IBM Corp., Armonk, NY).

## 3. Results

Table 1 shows the baseline characteristics of the participants according to their absolute ALM (low ALM vs. high ALM). The participants with low ALM had lower height, weight, BMI, waist circumference, ALM/Ht^2^, and ALM/BMI (P<0.05). No significant difference was observed in age, ALM/Wt, HbA1c, fasting glucose, HOMA-IR, SBP, DBP, hazard ratio, total cholesterol, triglycerides, HDL and LDL cholesterol, PAL, and leg power per weight between groups.

**Table 1:** Baseline characteristics of the study participants according to absolute ALM (N = 346).

	Low ALM			High ALM			
	mean	±	SD	mean	±	SD	P value
Age (y)	56.8	±	11.5	54.6	±	10.6	0.066
Height (cm)	153.7	±	5.1	159.9	±	4.7	<0.001
Weight (kg)	50.3	±	5.1	59.1	±	6.8	<0.001
BMI (kg/m^2^)	21.3	±	2.5	23.1	±	3.0	<0.001
Waist circumference (cm)	78.2	±	8.1	84.1	±	9.0	<0.001
ALM (kg)	15.1	±	0.8	17.5	±	1.1	<0.001
ALM/Ht^2^ (kg/m^2^)	6.4	±	0.4	6.8	±	0.5	<0.001
ALM/Wt (%)	30.2	±	2.5	29.8	±	2.8	0.108
ALM/BMI	0.717	±	0.076	0.764	±	0.092	<0.001
HbA1c (%)	5.5	±	0.5	5.4	±	0.4	0.299
Fasting glucose (mg/dL)	90.6	±	14.1	90.5	±	10.5	0.940
HOMA-IR	0.92	±	0.74	0.96	±	0.64	0.616
SBP (mmHg)	117.5	±	17.1	120.9	±	17.2	0.071
DBP (mmHg)	69.7	±	10.0	71.8	±	11.0	0.071
HR (bpm)	59.9	±	9.1	59.6	±	8.8	0.713
Total cholesterol (mg/dL)	219.5	±	34.4	216.7	±	36.0	0.461
Triglycerides (mg/dL)	84.7	±	45.9	85.3	±	45.5	0.901
HDL cholesterol (mg/dL)	72.6	±	16.0	69.4	±	16.8	0.071
LDL cholesterol (mg/dL)	129.5	±	29.9	129.8	±	31.7	0.924
PAL	1.61	±	0.12	1.61	±	0.14	0.773
Leg Power (W/kg)	14.9	±	4.2	14.9	±	3.8	0.930

SMI, skeletal muscle mass index; BMI, body mass index; ALM, appendicular lean mass; SBP, systolic blood pressure; DBP, diastolic blood pressure; HR, heart rate; HDL, high density lipoprotein; LDL, low density lipoprotein; PAL, physical activity level by using accelerometer.

Table 2 shows the baseline characteristics of the participants according to their ALM/Wt (low ALM/Wt vs. high ALM/Wt). The participants with low ALM/Wt had higher weight, BMI, waist circumference, HOMA-IR, SBP, DBP, hazard ratio, total cholesterol, and LDL cholesterol, as well as lower ALM/Wt, ALM/BMI, HDL cholesterol, physical activity level, and leg power per weight (P<0.05).

**Table 2:** Baseline characteristics of the study participants according to relative ALM against body weight (N = 346).

	Low ALM/Wt			High ALM/Wt			
	mean	±	SD	mean	±	SD	P value
Age (y)	56.2	±	9.8	55.4	±	12.3	0.475
Height (cm)	156.8	±	5.8	156.6	±	5.8	0.759
Weight (kg)	59.0	±	6.9	50.1	±	4.9	<0.001
BMI (kg/m^2^)	24.0	±	2.7	20.4	±	1.7	<0.001
Waist circumference (cm)	86.8	±	7.6	75.4	±	6.3	<0.001
ALM (kg)	16.4	±	1.5	16.1	±	1.5	0.094
ALM/Ht^2^ (kg/m^2^)	6.7	±	0.6	6.6	±	0.5	0.067
ALM/Wt (%)	27.9	±	1.5	32.1	±	1.6	<0.001
ALM/BMI	0.687	±	0.065	0.791	±	0.076	<0.001
HbA1c (%)	5.5	±	0.5	5.5	±	0.5	0.437
Fasting glucose (mg/dL)	91.1	±	11.9	90.0	±	13.0	0.403
HOMA-IR	1.12	±	0.88	0.76	±	0.37	<0.001
SBP (mmHg)	121.3	±	16.4	117.0	±	17.8	0.021
DBP (mmHg)	71.9	±	9.8	69.6	±	11.0	0.042
HR (bpm)	61.0	±	9.3	58.6	±	8.5	0.014
Total cholesterol (mg/dL)	223.1	±	34.6	213.3	±	35.2	0.009
Triglycerides (mg/dL)	95.1	±	50.1	74.9	±	38.3	<0.001
HDL cholesterol (mg/dL)	67.6	±	15.6	74.5	±	16.6	<0.001
LDL cholesterol (mg/dL)	136.1	±	29.6	123.3	±	30.6	<0.001
PAL	1.59	±	0.11	1.63	±	0.15	0.001
Leg Power (W/kg)	14.0	±	4.0	15.8	±	3.8	<0.001

SMI, skeletal muscle mass index; BMI, body mass index; ALM, appendicular lean mass; SBP, systolic blood pressure; DBP, diastolic blood pressure; HR, heart rate; HDL, high density lipoprotein; LDL, low density lipoprotein; PAL, physical activity level by using accelerometer.

Table 3 shows the baseline characteristics of the participants according to their ALM/Ht^2^ (low ALM/Ht^2^ vs. high ALM/Ht^2^). The participants with low ALM/Ht^2^ had lower age, weight, BMI, waist circumference, ALM, ALM/Ht2, HbA1c, fasting glucose, HOMA-IR, and SBP, as well as higher height, ALM/BMI, and HDL cholesterol (P<0.05).

**Table 3:** Baseline characteristics of the study participants according to relative ALM against square height (N = 346).

	Low ALM/Ht^2^			High ALM/Ht^2^			
	mean	±	SD	mean	±	SD	P value
Age (y)	54.2	±	10.9	57.4	±	11.2	0.008
Height (cm)	157.9	±	5.3	155.4	±	6.0	<0.001
Weight (kg)	51.5	±	5.9	57.6	±	7.6	<0.001
BMI (kg/m^2^)	20.6	±	1.9	23.8	±	2.8	<0.001
Waist circumference (cm)	77.3	±	7.8	84.8	±	8.6	<0.001
ALM (kg)	15.5	±	1.1	17.0	±	1.5	<0.001
ALM/Ht^2^ (kg/m^2^)	6.2	±	0.3	7.0	±	0.4	<0.001
ALM/Wt (%)	30.3	±	2.5	29.7	±	2.8	0.049
ALM/BMI	0.758	±	0.077	0.721	±	0.093	<0.001
HbA1c (%)	5.4	±	0.4	5.5	±	0.5	0.005
Fasting glucose (mg/dL)	88.8	±	12.2	92.4	±	12.5	0.007
HOMA-IR	0.83	±	0.46	1.05	±	0.86	0.003
SBP (mmHg)	115.3	±	16.2	123.0	±	17.4	<0.001
DBP (mmHg)	68.8	±	10.2	72.6	±	10.5	0.001
HR (bpm)	60.1	±	9.2	59.5	±	8.7	0.533
Total cholesterol (mg/dL)	218.0	±	35.4	218.3	±	35.0	0.931
Triglycerides (mg/dL)	81.2	±	44.9	88.7	±	46.2	0.127
HDL cholesterol (mg/dL)	73.2	±	16.3	68.9	±	16.4	0.014
LDL cholesterol (mg/dL)	128.1	±	31.4	131.2	±	30.1	0.343
PAL	1.62	±	0.13	1.61	±	0.14	0.481
Leg Power (W/kg)	15.2	±	4.1	14.5	±	3.9	0.100

SMI, skeletal muscle mass index; BMI, body mass index; ALM, appendicular lean mass; SBP, systolic blood pressure; DBP, diastolic blood pressure; HR, heart rate; HDL, high density lipoprotein; LDL, low density lipoprotein; PAL, physical activity level by using accelerometer.

**Fig 1:**
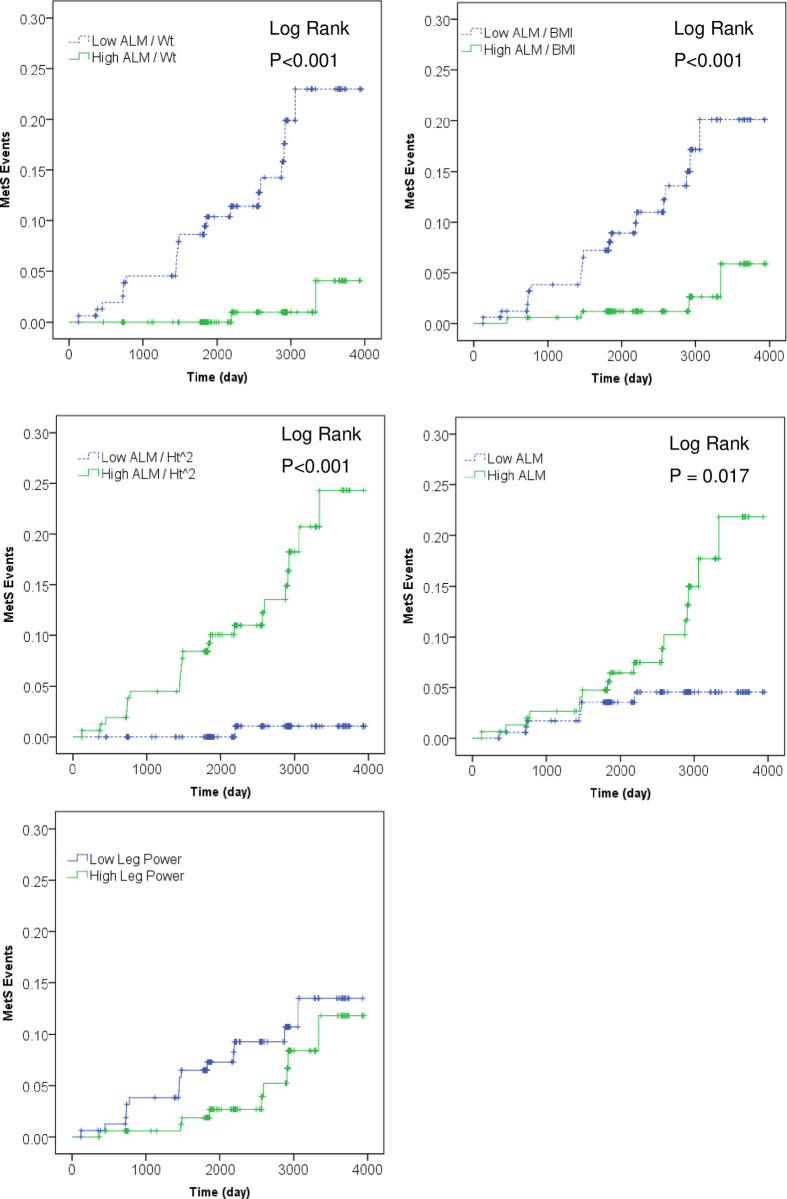
Kaplan-Meier curves for events of incident metabolic syndrome (MetS). Bold green line shows High ALM indices group, and dashed blue line shows Low ALM indices group.

Fig 1 shows the Kaplan-Meier curves for events MetS incidence according to baseline ALM indices. The participants with low ALM/Wt or ALM/BMI had significantly higher MetS incidence during the follow-up period (P<0.001). Furthermore, the participants with high ALM/Ht^2^ or absolute ALM had significantly higher MetS incidence during the follow-up period (P<0.001 and P = 0.017, respectively). Leg muscle power was not associated with MetS incidence (P = 0.143).

**Fig 2:**
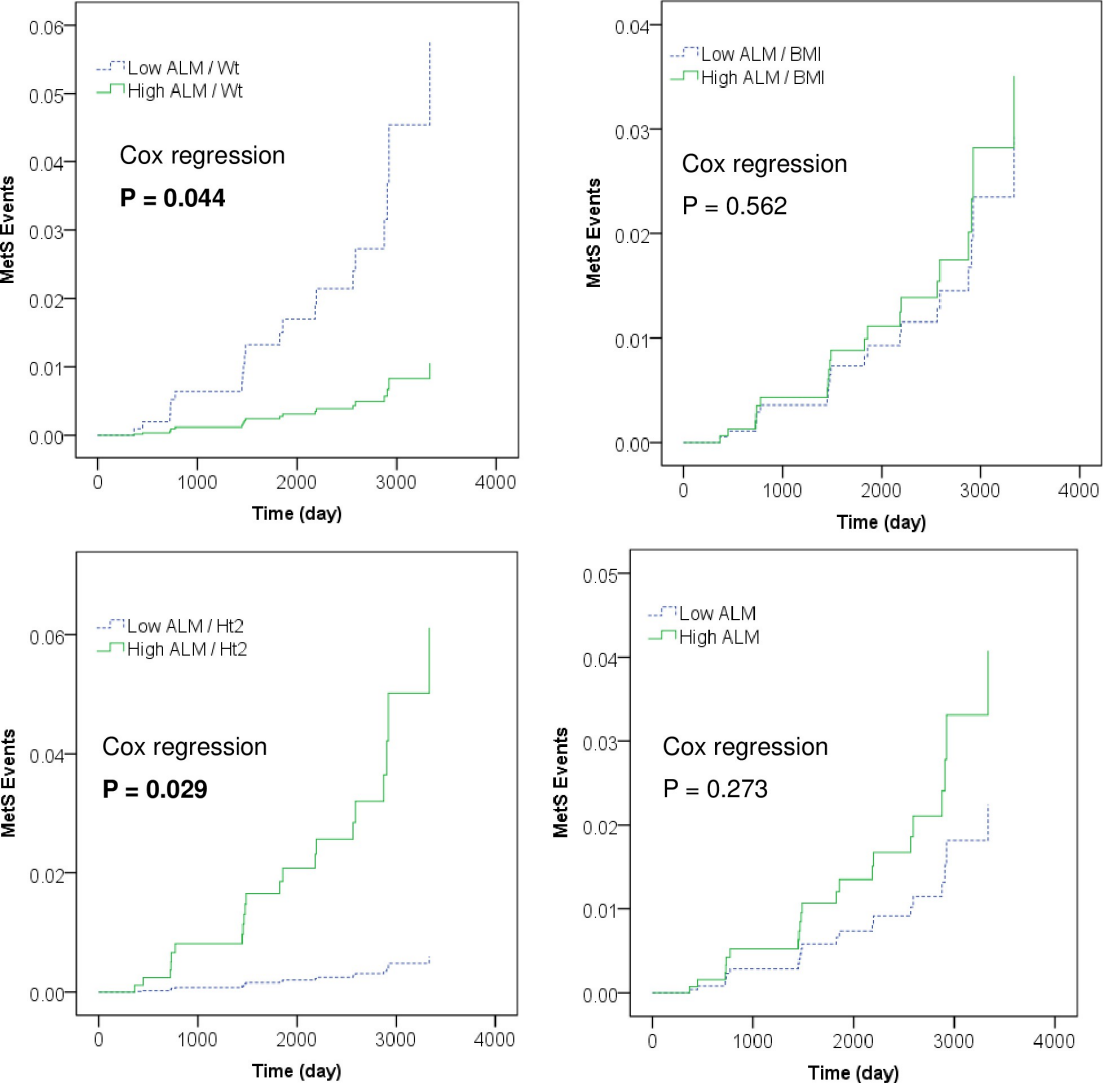
Cox proportional hazards model for events of incident metabolic syndrome (MetS). Bold green line shows High ALM indices group, and dashed blue line shows Low ALM indices group. Participants with low ALM/Wt showed significant association with an increased adjusted hazard ratio for MetS incidence compared with the participants with high ALM/Wt, after model 3 adjustment (see Table 4). In contrast, participants with high ALM/Ht2 showed significant association with an increased adjusted hazard ratio for MetS incidencecompared with the participants with low ALM/Ht2, after model 3 adjustment.

Cox proportional hazard regression analyses results are presented in Table 4. After model 2 adjustment, ALM/BMI and absolute ALM did not associate with MetS incidence (Fig 2). After model 3 adjustment, participants with low ALM/Wt showed significant association with an increased adjusted hazard ratio for MetS incidence [5.60 (95%CI: 1.04–30.0)] compared with the participants with high ALM/Wt (Fig 2). In addition, participants with high ALM/Ht^2^ showed significant association with an increased adjusted hazard ratio for MetS incidence [10.6 (95%CI: 1.27–89.1)] compared with the participants with low ALM/Ht^2^, after model 3 adjustment (Fig 2).

**Table 4:** Association between baseline ALM indices or leg muscle power and incidence of metabolic syndrome (Cox model) (N = 346).

	Model 1	Model 2	Model 3
	HR (95%CI)	HR (95%CI)	HR (95%CI)
High ALM/Wt (%)	**1 (ref)**	**1 (ref)**	**1 (ref)**
Low ALM/Wt (%)	**14.0 (3.29–59.6)**	**5.27 (1.08–25.8)**	**5.60 (1.04–30.0)**
	**P < 0.001**	**P = 0.040**	**P = 0.044**
High ALM/BMI	**1 (ref)**	1 (ref)	1 (ref)
Low ALM/BMI	**5.85 (2.00–17.13)**	1.57 (0.49–5.03)	0.83 (0.22–3.18)
	**P < 0.001**	P = 0.451	P = 0.787
High ALM/Ht2	**25.2 (3.40–186.6)**	**10.9 (1.40–84.2)**	**10.6 (1.27–89.1)**
Low ALM/Ht2	**1 (ref)**	**1 (ref)**	**1 (ref)**
	**P < 0.001**	**P = 0.022**	**P = 0.029**
High ALM	**2.80 (1.16–6.76)**	1.37 (0.51–3.72)	1.84 (0.62–5.45)
Low ALM	**1 (ref)**	1 (ref)	1 (ref)
	**P = 0.022**	P = 0.536	P = 0.273
High leg power	1 (ref)	1 (ref)	1 (ref)
Low leg power	1.84 (0.80–4.20)	1.12 (0.48–2.63)	0.75 (0.28–2.00)
	P = 0.149	P = 0.798	P = 0.562

SMI, skeletal muscle mass index; BMI, body mass index; ALM, appendicular lean mass; HR, hazard ratio; CI, confidence interval.

Model 1: crude

Model 2: Model 1 + further adjusted for age, obesity, waist circumference

Model 3: Model 2 + further adjusted for family history of diabetes, smoking status, physical activity level

When fat mass (FM) was entered in the Cox proportional hazard regression analyses with age, ALM/Wt, family history of diabetes, smoking status, and physical activity level, only age and FM had a significant AHR of MetS incidence (P<0.001), and ALM/Ht^2^ was no more a significant predictive variable (P = 0.714) (Table 5). ALM/Ht^2^ was also not a significant predictive variable (P = 0.412) for MetS incidence when FM was taken into account in the Cox regression model. In addition, percent body fat was entered in the Cox regression model as well as other variables, percent body fat was a significant predictor for MetS incidence (P<0.05).

**Table 5:** Cox regression models of ALM and fat mass for MetS incidence.

	Model 1		Model 2		Model 3	
	AHR	P	AHR	P	AHR	P
Age	1.11 (1.05–1.18)	<0.001	1.13 (1.06–1.20)	<0.001	1.11 (1.04–1.18)	0.003
ALM/Wt	0.31 (0.18–0.54)	<0.001	0.71 (0.28–1.84)	0.714	1.85 (0.47–7.28)	0.380
Fat mass			2.39 (1.08–5.29)	0.032	0.94 (0.29–3.10)	0.919
Percent body fat					8.22 (1.03–65.7)	0.047
	Model 4		Model 5		Model 6	
	AHR	P	AHR	P	AHR	P
Age	1.08 (1.03–1.14)	0.002	1.12 (1.06–1.19)	<0.001	1.11 (1.04–1.18)	0.002
ALM/Ht^2^	2.27 (1.39–3.71)	0.001	1.26 (0.72–2.20)	0.412	1.35 (0.76–2.41)	0.305
Fat mass			2.69 (1.60–4.53)	<0.001	0.81 (0.23–2.85)	0.738
Percent body fat					4.65 (1.08–20.0)	0.039

ALM/Wt, ALM/Ht^2^, Fat mass, Percent body fat are included in the models as Z-transformed values.

All models includes family history of diabetes, smoking status, physical activity level.

ALM, appendicular lean mass; AHR, adjusted hazard ratio.

## 4. Discussion

To the best of our knowledge, this is the first prospective study that examined the effect of fat mass or percent body fat on the association between relative or absolute ALM, leg muscle power, and MetS development. The major findings of this 7-year prospective study revealed that participants with low ALM/Wt showed significant association with increased adjusted hazard ratios for MetS incidence, compared with participants with high ALM/Wt, after model 3 adjustment. However, participants with high ALM/Ht^2^ showed significant association with increased adjusted hazard ratios for MetS incidence, compared with participants with low ALM/Ht^2^, after model 3 adjustment. In addition, any of ALM indices showed significant association with MetS incidence after fat mass or percent body fat was entered the Cox model.

SMM or ALM are strongly correlated with body size [32]. Thus, the European Working Group on Sarcopenia in Older People stated that “when quantifying muscle mass, the absolute level of SMM or ALM can be adjusted for body size in different ways, namely using height squared (ALM/height^2^), weight (ALM/Wt) or body mass index (ALM/BMI) [2].” Preferred adjustment has been a subject of debate. Janssen et al. [33] indicated that SMM/Wt is associated with functional impairment and disability in NHANES III participants aged 18 and older. Janssen [34] also indicated that SMM/Ht^2^ is associated with physical disability in participants aged 65 and older in the Cardiovascular Health Study (CHS) database. Furushima et al. [16] indicated that low ALM/Ht^2^ is associated with bone mineral density, but not with MetS variables, and that ALM/Wt is associated with MetS variables, but not with bone mineral density. Many previous studies also indicated that ALM/Wt is associated with MetS [12–16]. A recent study found a significant association between low ALM/Wt and MetS development in a 7-year retrospective study [35]. The results of the current study are consistent with these reports, and this is the first prospective study to examine the association between low ALM/Wt and MetS development, to the best of our knowledge. In contrast, high ALM/Ht^2^ showed significantly higher MetS incidence during the follow-up period, which is consistent with a recent cross-sectional study [36] and previous studies [10–17]. However, most of the previous studies did not take into account the effect of fat mass or percent body fat on the association between ALM and MetS development.

Results of this study clearly show that normalizing ALM to body weight and BMI leads to spurious interpretation, because the association is driven by body weight/fat. In Table 2, the group categorized as low ALM/Wt has the same amount of ALM as the High group, but 9 kg of weight more. Even when normalizing for height squared, the association of high ALM/Ht^2^ with MetS is driven by body fat: in Table 3, the group with high ALM/Ht^2^ has higher weight, BMI and waist circumference, all closely associated with MetS. These data are very useful to explain why the Cox models adjusted for percent body fat show that ALM is not an independent predictor of MetS and body fat should always be considered when investigating these relationships

The association between muscle strength or power and MetS has also been examined. Jurca et al. [10, 11] indicated that low muscular strength index computed by combining the one-repetition maximum score for bench press and leg press expressed as weight lifted per kilogram body weight was significantly associated with high MetS prevalence. Recently, Zhang et al. [17] examined the association between MetS prevalence and absolute or relative values of muscle strength in women. They concluded that prevalence increased with low relative grip strength and leg strength (per kilogram body weight). Conversely, low absolute muscle strength was associated with low MetS prevalence [17]. The current longitudinal study showed a significant association between low relative muscle power and MeTS development, but the association was not more significant after adjustment using model 3.

This study has several limitations. First, because of limited sample size, we could not test many adjusting variables in this study. There may be possible confounders between low relative SMM and MetS development. Second, muscle quality and composition, including fat infiltration [37, 38], fibrosis [39, 40], and relative expansion of extracellular compartments [3, 41, 42], are important in muscle tissue assessment. However, we could only assess ALM in this study. Further studies are needed to address this issue. Third, recently, BIA has been used in many studies to assess ALM, but the BIA method is a secondary indirect method to estimate body composition [43]. BIA is possibly influenced by edema, exercise, and circadian and seasonal variations [44–46]. Although BIA was measured in the morning without any exercise and fasting state in this study, seasonal variations may still affect the current results. Other limitations of this study include important potential confounders not taken into account, such as changes in weight and physical activity during the follow-up period.

## 5. Conclusions

In conclusion, our results show that ALM/Wt is negatively associated future development of MetS in Japanese women. In contrast, relative ALM/Ht^2^ was positively associated the future development of MetS in Japanese women. Absolute ALM did not associate with the future development of MetS after adjusting for age, obesity, waist circumference, family history of diabetes, smoking status, and physical activity level. The relationship between SMM and MetS development is more complex than previously thought. To resolve this issue, a model that takes into account the fat mass and fat-free mass relationship must be constructed. Interestingly, both ALM/Ht^2^ and ALM/Wt were not significant predictive variables for MetS development when fat mass or percent body fat was taken into account in the Cox model. At the very least, the results of this study underscore the importance of body composition measurements in that percent body fat is associated with MetS development.
